# Effect of 900 MHz Electromagnetic Radiation on the Induction of ROS in Human Peripheral Blood Mononuclear Cells

**Published:** 2015-09-01

**Authors:** E. Kazemi, S. M. J. Mortazavi, A. Ali-Ghanbari, S. Sharifzadeh, R. Ranjbaran, Z. Mostafavi-pour, F. Zal, M. Haghani

**Affiliations:** 1 Radiobiology department, School of paramedical sciences, Shiraz University of Medical Sciences, Shiraz, Iran; 2 Medical Physics Department, School of Medicine, Shiraz University of Medical Sciences, Shiraz, Iran; 3 The Center for Research on Protection against Ionizing and Non-ionizing Radiation, School of Paramedical Sciences, Shiraz University of Medical Sciences, Shiraz, Iran; 4 Telecommunication Department, School of Engineering, Shiraz University, Shiraz, Iran; 5 Diagnostic Laboratory Sciences and Technology Research Center, School of Paramedical Sciences, Shiraz University of Medical Sciences, Shiraz, Iran; 6 Biochemistry Department, School of Medicine, Shiraz University of Medical Sciences, Shiraz, Iran

**Keywords:** Electromagnetic Radiation, Radiofrequency (RF), Mobile phone, Reactive Oxygen Species (ROS), Human Peripheral Blood Mononuclear Cells

## Abstract

**Background:**

Despite numerous studies over a decade, it still remains controversial about the biological effects of RF EMF emitted by mobile phone telephony.

**Objective:**

Here we investigated the effect of 900 MHz GSM on the induction of oxidative stress and the level of intracellular reactive oxygen species (ROS) in human mononuclear cells, monocytes and lymphocytes as defence system cells.

**Method:**

6 ml Peripheral Blood samples were obtained from 13 healthy volunteers (21-30 year-old). Each sample was devided into 2 groups: one was exposed RF radiation emitted from a mobile phone simulator for 2 hour and the other used as control group which was not exposed to any fields. After that, mononuclear cells were isolated from peripheral blood by density gradient centrifugation in Ficoll-Paque. The intracellular ROS content in monocytes and lymphocytes was measured by the CM-H2DCFDA fluorescence probe using flowcytometry technique.

**Results:**

Our results showed significant increase in  ROS production after exposure in population rich in monocytes. This effect was not significant in population rich in lymphocytes in comparison with non exposed cells.

**Conclusion:**

The results obtained in this study clearly showed the oxidative stress induction capability of RF electromagnetic field in the portion of PBMCs mostly in monocytes, like the case of exposure to micro organisms, although the advantages or disadvantages of this effect should be evaluated.

## Introduction

The interest in evaluation of health effects induced by non-ionizing radiation exposure of radiofrequency (RF) electromagnetic fields has largely increased in the last decades, mostly motivated by the expanding use of mobile phones telecommunications worldwide. Mobile networks utilize different frequencies in different parts of the world. Global System for Mobile Communications (GSM) networks are in 900, 1800 MHz bands in the range of RF waves. An increase of temperature in culture medium of RF-EMF exposed cells has been observed at very high Specific Absorption Rate (SAR) levels only, so the thermal effect of RF emitted by mobile phones with SAR safety limit of 2 W/kg is negligible as it’s inadequate to cause detectable temperature increase or induce thermal effects[1]; meanwhile, several studies have indicated that low-power RF, may elicit a biological effect in target cells or tissues[2, 3]. However it is not clear whether or not these biological effects lead to adverse health effects.

A considerable proportion of studies have investigated the “non-thermal” effects of RF in the cells and tissues, showing that this effect is mediated by generation of Reactive Oxygen Species (ROS) like hydroxyl radical °OH, hydrogen peroxide H2O2[4]. It is reported that EMFs in their entire frequency spectrum (low to high) induce an increase in oxidative stress and oxygen-free radicals in many experimental systems (including plants) and in man[5]. The first step in generation of ROS by RF is mediated in plasma membrane by NADH oxidase. Subsequently ROS activates matrix metalloproteases (MMP), thereby initiating intracellular signaling cascades to warn the nucleus about the presence of external stimulation which in turn changes in transcription and protein expression observed after RF exposure[6]. These molecules are so reactive and oxidize other cellular components such as proteins, lipids and DNA[7]. However cells have the antioxidant system to scavenge these species but in the case of overwhelming amounts of ROS and underproduction of cellular antioxidant, various degrees of oxidative stress occurs which cause impairments in the mentioned components and their normal actions. This happens when some diseases such as diabetes or cancer occur[8, 9]. However, release of ROS is an important function in some cells, like monocytes and macrophages, during the attack of microorganisms to the body[10, 11]. So ROSs are playing a dual role as both harmful and beneficial to the living systems.

Oxidative stress induced by RF production in special tissues and cells has been reported, indicating that electromagnetic fields put stress on living cells[12]. On the other hand, some studies were done to improve the neutralizing effect of the presence of antioxidants during the RF exposure in vivo and in vitro[13, 14]. These reports show that RF can induce oxidative stress in a way that antioxidants can lower this effect. All reports improve the oxidative stress induction of RF hypothesis.

In the present study, we used human Peripheral Blood Mononuclear Cells (PBMC) which are used in many previous studies on the bioeffects induced by EMFs in the range of ELF and RF. Also, some researchers believe that PBMCs are proper cell types used to evaluate the effect of EMF under special stimuli, representing the major immune response in humans[15, 16].

Over the past years, our laboratory has focused on studying the health effects of exposure of laboratory animals and humans to some common and/or occupational sources of electromagnetic fields such as mobile phones[17-24] and their base stations[25], mobile phone jammers[26], laptop computers[27], radar[28], dentistry cavitrons[29]. The aim of this study was to investigate whether 2 hour exposure of human mononuclear cells can change reactive oxygen species release or not.

### Donors and Blood Samples

The study was approved by the Experimentation Ethics Committee of Shiraz University of Medical Sciences prior to commencing. Informed consent was obtained from human subjects included in the study. The blood was considered healthy following routine laboratory analysis. Peripheral Blood samples were obtained from 13 healthy volunteers (21-30 year-old) in the volume of 12 ml who had not been under any therapeutic or occupational ionizing radiation and were collected into heparinized tubes. Each sample from each volunteer was divided into two groups: one applied as a control group and the other as the exposed group. There was also a positive control group which was exposed to 0.1 µl of H2O2 for 2 hours.

### Reagent

PBS were from Gibco; CM-H2DCFDA reagent from Molecular Probes C6827, Invitrogen Co; dimethyl sulfoxide (DMSO) from sigma; Ficoll-Paque from sigma. 

### Exposure system

A GSM mobile phone simulator was used for radiofrequency irradiation. The frequency was adjustable from 800 to 1800 MHz but in this experiment 900 MHz radiation (a wavelength of about 33.4 cm) similar to GSM mobile phone was used. Amplitude was digitally modulated with 217Hz frequency and duty cycle was 0.125 (the signal was switched ON for 463 micro seconds periodically with a rate of 217 Hz. The power was adjustable from zero to 3 Watts but we fixed it at 2 W during the exposure. The signal bandwith was 200kHz (similar to GSM mobile phone channels). To achieve a homogeneous exposure of peripheral blood cells, a rectangular waveguide was also used in this project. It was made up of aluminum in which the total resistive loss was negligible. A place with maximum electric and magnetic fields in the waveguide was specified to guarantee the relatively homogeneous exposure of samples. The exposure groups of peripheral blood samples in 6 well dishes were put in the mentioned place of waveguide. Waves emitted by the simulator were transferred to the waveguide by a co-axial wire. The increase of temperature was not more than 0.1 °C and the time duration of exposure was 2 hours.

After the exposure, the mononuclear cells of each sample, control and exposure, were isolated from the peripheral blood by density gradient centrifugation in Ficoll-Paque (1800 rpm-15 min) and washed in PBS. Viability of the cells was checked to ascertain whether the cells examined for ROS production are in maximum viability or not.

### Assay of Intracellular ROS

A fresh stock solution of CM-H2DCFDA (5Mm) was prepared in DMSO and diluted to a final concentration of 1 µM in 1×PBS. 50 µL of working solution of fluorochrome marker CM-H2DCFDA (final working concentration adjusted to 2.5µg / 50 µL) was added to the cells, kept in 37ºC for 30 minutes in dark,  and then they  were washed with 1×PBS twice. Finally cell-associated mean fluorescent intensity was measured by flow cytometry in FL1 channel excitation and emission wavelengths were 488 and 525 nm, respectively. 

The intracellular ROS content was measured by DCFH oxidation. The CM-H2DCFDA reagent passively diffused into cells. It was hydrolysed by intracellular esterase to liberate 2´ -7´ -dichlorofluoressein, which, during the reaction with oxidizing species, yields a highly fluorescent compound 2´-7´-dichlorofluorescein (DCF) that is trapped inside the cells. DCF is highly fluorescent and indirectly shows the ROS production in the cell. 

**Figure 1:**
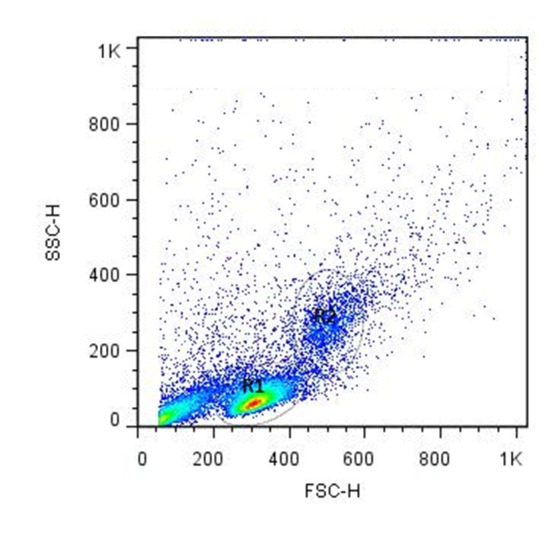
Dot plot cytogram of peripheral blood mononuclear cells. They are gated based on side scatter (SSC) and forward scatter (FSC). R1 is population enriched of lymphocytes. R2 is population enriched with monocytes.

The amount of ROS production was evaluated as mean flourcent intensity in two separated population enriched with lymphocytes and monocytes. According to dot plot cytogram in Figure 1, peripheral blood mononuclear cells, were gated on the basis of forward and side scattering in flowcytometry. 5×10[4] cells were evaluated as reactive oxygen species.

## Results

**Figure 2:**
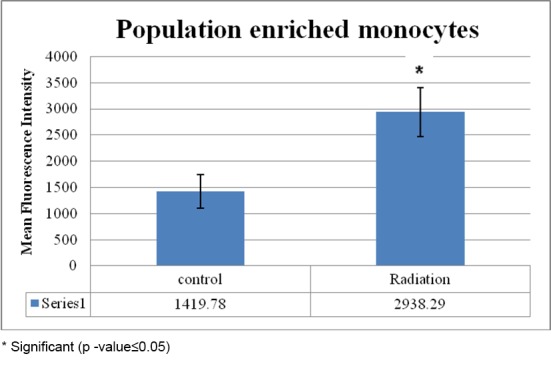
DCF fluorescence measurement of ROS in the cell population enriched with human monocytes in the control and exposure groups for 2 hours

Figure 2 presents the effect of 2 hour exposure of the population enriched with human monocytes to microwave EMF on the ROS generation. This population were isolated from PBMCs. An increase in ROS synthesis in exposure groups versus control groups was observed. ROS generation is expressed as mean fluorescence intensity of CM-H2DCFDA dye detected by flow cytometry.

**Figure 3:**
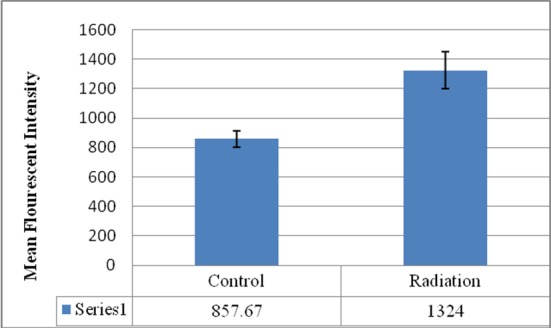
DCF fluorescence measurement of ROS in human lymphocytes in the control and exposure groups for 2 hours

Figure 3 displays the ROS generation in human lymphocytes of the two groups, control and exposure, expressed as mean fluorescence intensity. The difference of mean fluorescence intensity between the two groups was not significant.

**Figure 4:**
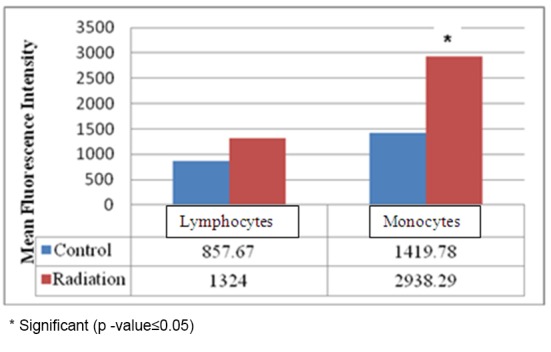
Comparison of mean fluorescence intensity between human lymphocytes and population enriched with monocytes isolated from PBMCs

According to Figure 4, ROS content in the unexposed group of cell population enriched monocytes was more than that in human lymphocytes and two hour exposure of 900 MHz EMF increased the ROS generation in the population enriched with human monocytes versus lymphocytes. The increase in ROS synthesis in the lymphocytes after 2 hour exposure was not significant.

**Figure 5:**
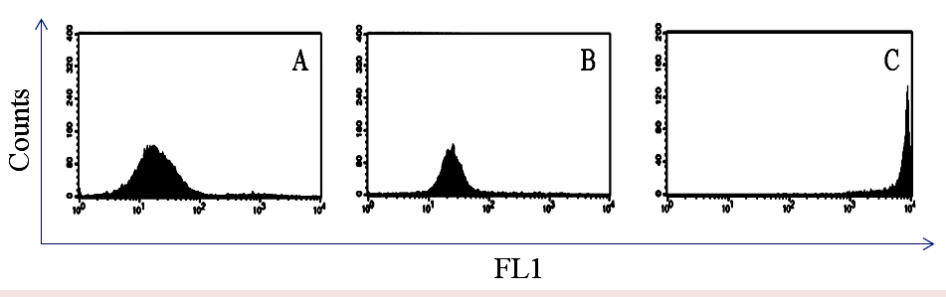
Flow cytometry evaluation of mean fluorescence intensity of DCF in the control group of lymphocytes not exposed to EMF (A), 900 MHz exposure group of lymphocytes (B) and lymphocytes exposed to H2O2 (0.2µM) for two hours (C). Two hour exposure of lymphocytes to H2O2 increased the ROS production.

**Figure 6:**
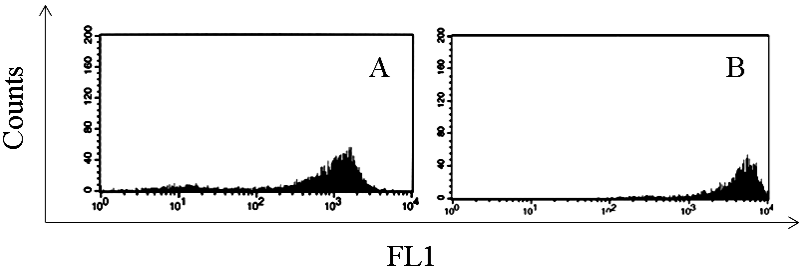
Flow cytometry evaluation of mean fluorescence intensity of DCF in the control group of the population enriched with human monocytes not exposed to EMF (A), 900 MHz exposure group of the population enriched with human monocytes (B).

Figure 5 is a flow cytometric histogram of DCF intensity in lymphocyte samples in control, exposure to 900 MHz and H2O2 groups. According to this histogram, the DCF intensity in group exposured to H2O2 is high. The histogram of DCF intensity in exposed and control groups of population with too much monocytes is in Figure 6 which shows the significant DCF intensity after exposure.

## Discussion

The aim of the current study was to verify if 900 MHz EMF with 2W power could induce oxidative stress in human PBMCs. The endpoint was the amount of ROS production before and after exposure. The results did not show any difference in ROS production after exposure to the 900 MHz RF EMF in the lymphocyte rich population samples used. Besides, ROS production in the monocytes rich population was increased after the exposure. As shown in recent studies, the non-thermal effect of EMF exposure is related to its ability in cell stimulation which leads to ROS production. Induction of oxidative stress was shown in some studies. Stankiewicz et al reported that human lymphocytes and monocytes accelerate their metabolic activity under additional stimulus created by exposure to 900 MHz GSM signal[30]. Zmyślony et al. reported increased ROS production after the exposure of RF (5-15 min)  in the presence of 10 µgr/ml FeCl2 in the rat lymphocyte cells but not in the case of RF alone[31]. Lantow et al used human Mono Mac 6 (primary human lymphocytes) and K562 cells and exposed them to 1800 MHz, GSM. Exposure was continuous (5 min ON-5min OFF). But no oxidative stress was observed[32]. Likewise, Hook et al reported no differences in oxidative stress parameter in macrophage J774.16 mouse cells after 20-22 hr exposure to 847.74 MHz[33]. Human monocytes displayed a significant increase in ROS production due to synergistic effect of PMA[34].

Luukkonen et al. investigated the effect of 872 MHz on ROS production in SH-SY5Y (human neuroblastoma) cell line. It was shown that ROS production was higher in the cells exposed to CW RF radiation 30 and 60 min after the exposure. But GSM-like modulated signal did not show this effect[35].  But Höytö et al showed a significant lipid peroxidation (product of oxidative stress) induction after exposure to GSM modulated signal in the mouse L929 fibroblast cells and SH-SY5Y cell[36]. In summary, oxidative stress induction is not observed in all cell types and it shows the differences in sensitivity of cells to EMF exposure.

Isolated PBMC are a mixture of monocytes and various subtypes of lymphocytes, including T, B, and natural killer cells. Monocytes, which belong to the group of antigen-presenting cells (APC) and diverse T lymphocytes (e.g., TCD4 helper-inducer and CD4, CD25 T-regulatory cells) are the main cellular elements that determine initiation and development of immune response. Monocytes kill microorganisms by lysosomal enzymes as well as oxidative burst that lead to the generation of ROS and NO. These componants play an important role in bactericidal activity. Also human blood monocytes release large amounts of ROS when freshly explanted and challenged with suitable immunologic, pharmacologic, or particular stimuli[37]. During an infective process in the body, circulating blood monocytes migrate from the vessels into extravascular compartment. In the tissue, monocytes differenciate into macrophages; they lose their ability to replicate but enhance their properties that allow them to participate in the inflammatory and immune response[38]. Macrophages are activated by stimulation  like lipopolysaccharides (LPO) in the cell wall of gram negative bacteria or cytokine IFN-γ released from T-cells and natural killing cells (NK cells). The percentages of NK cells in mononuclear cells of blood and spleen is about 5-20% in the form of large lymphocytes with numerous granules. Activated macroghages phagocyte the microorganism proceeding the integration with lysosome and formation of phagolysosome where the microorganisms are eliminated by reactive oxygen and nitrogen species[39, 40]. Both group of cells (monocytes and T-lymphocytes) which participate in immune response, are present in natural proportions in the population of PBMC and their functional state is proportional to produced cytokines (e.g., IL-1β, IL-1ra, TNF-α, IL-2, IL-10, IFN- γ)[41]. According to the results in our experiment, EMF may induce T cell and NK cells to produce IFN-γ that leads to the formation of ROS in monocytes.

A substantial evidence exists for the bactericidal cell activity of hydroxyl radical derived from leukocytes[42]. In addition, there are some reports revealing that hydroxyl radical scavengers inhibit the bactericidal activity in vivo and in vitro or superoxide dismutase and catalase (antioxidant enzymes) inhibit this activity in the phagocytes[43]. Some reports investigated this function under exposure to ELF EMF. Simkó et al showed the activation of murine macrophages when exposed to 50 Hz for 45 min[44]. Rollwitz et al reported the increasing production of superoxide radicals in differentiated macrophages exposed to ELF[45].

It can be concluded that EMF exposure can stimulate the monocytes like in the case of exposure to a microorganism. Probably, this effect is beneficial in the case of exposure to microorganism which EMF stimulate monocytes to start invading pathogen. So it is possible to conclude the immunotropic potency of EMF in stimulating the bactericidal activity of the monocytes. 

According to the results obtained in this study, it is concluded that 900 MHz electromagnetic field may stimulate the monocytes to generate reactive oxygen species but it was not seen in the lymphocytes. However, this result is scanty to come to the conclusion whether this effect is beneficial or not. So this topic deserves further investigations, paying attention to the role of RF exposure on immunity in the case of co-exposure to microorganisms. 
